# Saliva-derived transcriptomic signature for gastric cancer detection using machine learning and leveraging publicly available datasets

**DOI:** 10.1038/s41598-025-96864-0

**Published:** 2025-05-27

**Authors:** Catarina Lopes, Andreia Brandão, Manuel R. Teixeira, Mário Dinis-Ribeiro, Carina Pereira

**Affiliations:** 1 Precancerous Lesions and Early Cancer Management Group, Research Center of IPO Porto (CI-IPOP)/CI-IPOP@RISE (Health Research Group), Portuguese Institute of Oncology of Porto (IPO Porto)/Porto Comprehensive Cancer Center Raquel Seruca (Porto.CCC), Porto, Portugal; 2 Center for Health Technology and Services Research (CINTESIS@RISE), University of Porto, Porto, Portugal; 3 ICBAS – School of Medicine and Biomedical Sciences, University of Porto, Porto, Portugal; 4 Cancer Genetics Group, Research Center of IPO Porto (CI-IPOP)/CI-IPOP@RISE (Health Research Group), Portuguese Institute of Oncology of Porto (IPO Porto)/Porto Comprehensive Cancer Center Raquel Seruca (Porto.CCC), Porto, Portugal; 5 Department of Laboratory Genetics, Portuguese Institute of Oncology of Porto, Porto, Portugal; 6 Department of Gastroenterology, Portuguese Institute of Oncology of Porto, Porto, Portugal

**Keywords:** Early screening, Salivaomics, Liquid biopsies, Biomarkers, Bioinformatics, Cancer, Cancer screening, Gastrointestinal cancer, Tumour biomarkers

## Abstract

Saliva, a non-invasive, self-collected liquid biopsy, holds promise for early gastric cancer (GC) screening. This study aims to assess the potential of saliva as a proxy for malignant gastric transformation and its diagnostic value through transcriptomic profiling. Leveraging transcriptomic data from the Gene Expression Omnibus (GEO), we constructed and validated predictive models through machine learning algorithms within the tidymodels framework. Tissue-based models were validated on independent tissue datasets, and subsequently applied to saliva. Additionally, an independent saliva-derived model was created and evaluated using sensitivity, specificity, accuracy, area under the curve (AUC), and likelihood ratio (LR) metrics. Tissue-derived models demonstrated excellent performance, with AUC values exceeding 0.9, but did not translate effectively to saliva, suggesting distinct molecular landscapes between tissue and saliva in GC. The saliva-specific model using support vector machine (SVM) achieved the highest performance, with an AUC of 0.87 (95% CI 0.72–0.97), a sensitivity of 0.79 (95% CI 0.58–0.95) and a specificity of 0.70 (95% CI 0.40–0.90). While saliva may not mirror tissue gene expression profile, it represents a promising non-invasive predictive tool for the early detection of GC. Further research is warranted to optimize saliva-derived molecular signatures, increasing their sensitivity and specificity for early cancer detection and advance the use of liquid biopsies in personalized medicine for improved screening, diagnostic and prognostic capabilities.

**Supplementary Information:**

The online version contains supplementary material available at 10.1038/s41598-025-96864-0.

## Introduction

The EU Mission on Cancer set the challenge of improving the lives of more than three million people by 2030 through prevention, curing, and improving the quality of life for those affected by cancer^1^. Early detection through screening offers the best opportunity to overcome cancer^2^. In 2022, Europe’s Beating Cancer Plan advocated the expansion of current screening programs to encompass prostate, lung, and gastric cancer (GC) in countries and regions with high incidence and mortality rates^3^.

Current strategies for detecting GC involve minimally invasive gastrointestinal (GI) endoscopy, which is considered the gold standard for diagnosing the early and curable stages of GC and significantly enhances the survival and quality of life of patients^4,5^. However, as a standalone screening procedure, GI endoscopy is suboptimal in Western countries because of the paucity of adherence and cost-effectiveness^6,7^. In the era of personalized medicine, these challenges could be overcome through better risk assessment and patient stratification. This could be achieved by identifying and validating novel, preferably non-invasive, biomarkers with superior diagnostic performance compared with currently available tools. Such advancements offer the promise of tailored and more effective screening approaches.

Currently, serologic assessments of pepsinogen I/II, gastrin-17, and anti-*Helicobacter pylori* antibodies are recommended for identifying patients with atrophic gastritis, a precancerous condition, who should undergo endoscopy in Western countries^8–10^. However, this approach involves drawing blood, requiring the involvement of a health care provider and a clinical or laboratory setting.

Saliva emerges as an appealing alternative for non-invasive biomarker detection. It is accessible, safe, less costly, easy to store and collect, and does not require professional assistance, achieving a 70–95% compliance rate without added incentives - higher than conventional blood collection methods^11^. Furthermore, saliva can be repeatedly self-collected at home, which is advantageous for longitudinal monitoring and screening programs. Often referred to as a “mirror of body health”, saliva carries a diverse array of biomarkers, including lectins, RNAs, proteins, and bacteria, implicated in atrophic gastritis and GC detection^12,13^. Although it is not in direct contact with the stomach, systemic circulation and glandular secretions enable saliva to capture molecular signals reflective of pathological changes elsewhere in the body^14,15^. The successful use of saliva in detecting cancer biomarkers, such as head and neck^16^, lung^17^, pancreatic^18^, and even gastric cancer^19^, further highlights its potential in cancer screening. Therefore, saliva-based diagnostics could offer a scalable and patient-friendly approach to improve the timely early detection of GC and minimize inequalities in access to healthcare systems. However, can this non-invasive liquid biopsy be considered a proxy for malignant stomach transformation?

The continuous innovation of high-throughput sequencing technology coupled with advanced computational pipelines has allowed researchers to explore the human genome, epigenome, proteome, and transcriptome landscape synthetically. The burgeoning field of salivaomics focuses on the integrative study of saliva and all its constituents, taking advantage of omics technologies, which have revolutionized the identification and validation of disease biomarkers^14^. Leveraging publicly available data from the Gene Expression Omnibus (GEO), our study aimed to assess saliva as a non-invasive predictive tool for detecting GC. Furthermore, by comparing transcriptomic profiles of saliva and gastric tissue, we sought to understand whether the liquid biopsy reflects tissue changes associated with GC carcinogenesis. Predictive models based on differentially expressed genes (DEGs) from both tissue and saliva datasets were developed. While tissue-based models demonstrated strong performance in the initial validation, the suboptimal results on the saliva dataset suggested that saliva does not directly mirror the transcriptomic dysregulation observed in gastric tissue, but could saliva represent a liquid biopsy for personalized GC screening and early detection? Ultimately, we were able to establish a robust signature for non-invasive GC diagnosis, validating saliva as a reliable proxy for malignant transformation in the stomach.

## Methods

### Study design and microarray data sources

**Fig. 1:**
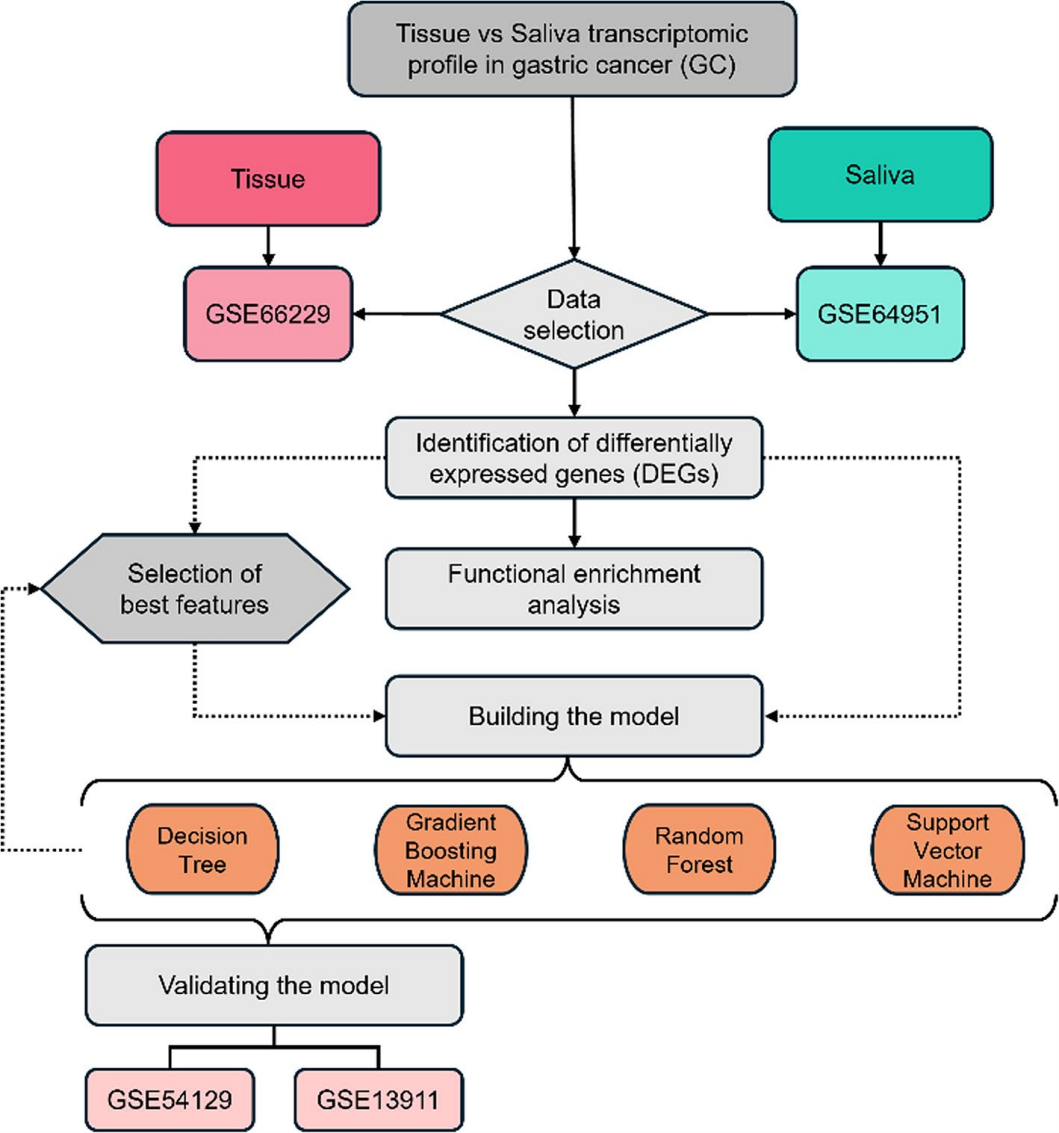
Overview of the study design.

Figure 1 provides an overview of the study design. Briefly, the process began with the selection of relevant data, followed by the discovery phase, where DEGs were identified. Functional enrichment analysis was performed to explore the biological significance of these genes, and the most important features were selected for further analysis. These selected features were then used to build machine learning models to predict GC based on gene expression patters in both tissue and saliva. Finally, the models developed from tissue data were validated using independent datasets to confirm their robustness and generalizability.

To assess saliva as a proxy of stomach malignant transformation, CEL files of two microarray datasets originating from the Affymetrix Human Genome U133 Plus 2.0 Array [HG-U133_Plus_2], which covers over 38 500 genes, were downloaded from GEO^20^. The tissue dataset (GSE66229) included tumor samples from 300 Korean patients with GC who underwent total or subtotal gastrectomy and 100 patient-matched normal tissues. Additional clinical and demographic information regarding the tissue sample cohort has been previously described^21^. The saliva dataset (GSE64951) included samples from Korean individuals, 63 GC patients and 31 controls whose RNA was extracted from the saliva supernatant^20^. Both datasets were obtained from the GPL570 platform and were chosen based on available datasets. Specifically, the saliva dataset was the sole comprehensive source of raw data available in GEO and the tissue-derived dataset gathered the most extensive patient set.

To further validate the tumor-derived models generalizability, external validation was conducting using independent datasets, GSE54129 dataset, which includes samples from 111 GC patients and 21 healthy controls from China, and GSE13911, which gathers samples from 38 GC patients and 31 adjacent normal tissues from Italy.

### Identification of differentially expressed genes associated with GC and functional enrichment analysis

Differential gene expression analysis was performed independently for each dataset via the “limma” R package to identify dysregulated genes between diseased and normal states^22^. The median expression level was used as a cutoff to filter genes with low expression. False discovery rate (FDR) analysis was performed to correct for multiple testing^23^. The thresholds for differential gene expression identification were set at an absolute value of log2-fold change greater than 1 (|logFC| > 1) and an adjusted P value less than 0.05 (adj. P value < 0.05), where logFC > 1 indicates upregulated genes and logFC < -1 indicates downregulated genes. Volcano plots and heatmaps were constructed to display the results of differential gene expression. Functional enrichment analysis was conducted to identify biological pathways and gene sets significantly associated with GC via “clusterProfiler” using gene set enrichment analysis (GSEA) and visualization of enrichment results was carried out using the “DOSE” and “rrvgo” R software packages^24–26^.

### Preprocessing and feature selection

To mitigate potential biases, all datasets underwent the same normalization procedures. Background correction, normalization, and expression calculation were performed on the tissue- and saliva-derived microarray data, which were first normalized using the robust multichip average (RMA) method from the “affy” R package, ensuring that the datasets were appropriately scaled and centred for consistency and reliability^27^. The datasets were subsequently filtered to retain only the differentially expressed genes (DEGs). The resulting gene expression values were then used as input features for training and evaluating the machine learning models. The tidymodels framework was utilized to conduct preprocessing, feature selection, and model building, including tuning, and evaluation^28^. The data were split into training and testing sets using the “rsample” package, with 70% of the data allocated to training and 30% allocated to testing. Stratified sampling was used to ensure that class distribution of the target variable was preserved in both sets. The “recipes” package was used to handle missing values and remove highly correlated features with a threshold of 0.85. Additionally, the “themis” package was applied to balance class distribution of the target variable in the training set through downsampling, to minimize the impact of class imbalance bias.

For feature selection, random forest (RF), gradient boosting machine (GBM), specifically eXtreme Gradient Boosting (XGBoost) and decision tree (DT) methods were employed. Each model was specified via the “parsnip” package within tidymodels, and key parameters were tuned to optimize performance. A resampling strategy was implemented, dividing the training data into 10 folds and repeating the process five times to ensure robust estimation of model performance. A grid search approach was used for hyperparameter tuning, with up to 10 parameter candidates for each model, using the “tune” and “dials” packages. Feature selection was performed prioritizing the best molecular models importance scores of all three algorithms using the “vip” package. Specifically, the top seven genes were selected considering their high-ranking importance scores in both RF and GBM models. Additionally, two of these genes also demonstrated high importance in the DT model. This approach provided a robust selection of DEGs with strong predictive relevance across multiple algorithms. The top genes were selected for further model building to reduce dimensionality and improve model performance.

### Final model building and evaluation

The final models, which employed RF, GBM (XGBoost), DT, and, additionally, Support Vector Machine (SVM), were built using the selected top genes. The results from the grid search were summarized and analysed to rank the models based on their performance metric. The best-performing model was selected based on the highest area under the receiver operating characteristic (ROC) curve (AUC) values, along with other supportive metrics, namely sensitivity, specificity, accuracy, positive predictive value (PPV) and negative predictive value (NPV), obtained using the “yardstick” package. ROC curves were plotted using the “pROC” package^29^. The final models built on the tissue training data, were directly applied to the external datasets GSE54129 and GSE13911. Performance was evaluated using the same metrics (AUC, sensitivity, specificity, accuracy, PPV, NPV) to assess the models applicability across populations.

### Statistical analysis

All data processing and analysis were performed in RStudio (version 2024.04.2.764) using R software (version 4.3.2). All the statistical P values were two-sided, and *P* < 0.05 or adj. *P* < 0.05 was considered to indicate statistical significance. The R code developed in-house for this study is available upon reasonable request. A comprehensive list of all R packages used, along with their versions and sources is displayed in Table S1.

## Results

### Saliva does not mirror the tumor-derived transcriptomic profile in the stomach

**Fig. 2:**
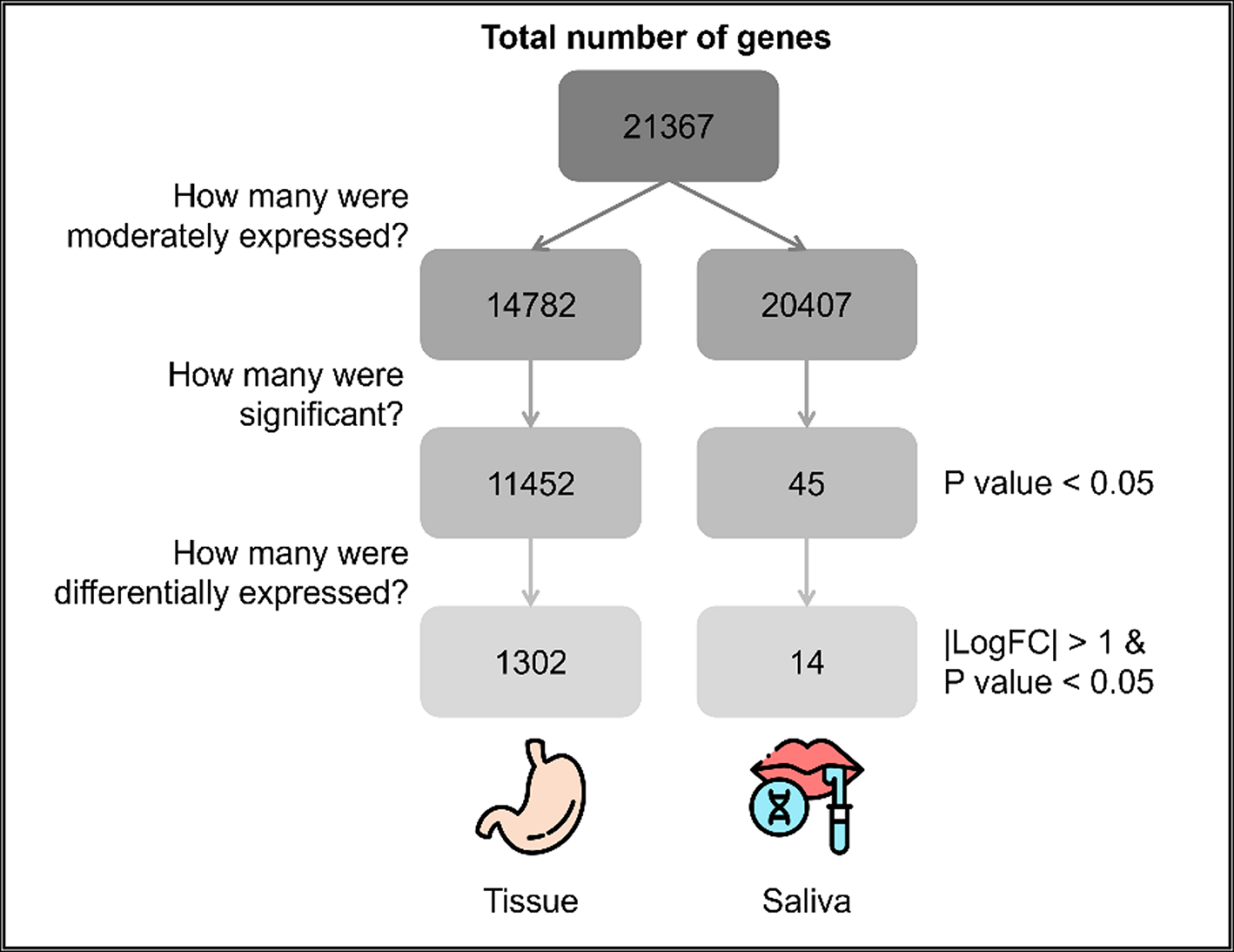
Number of tissue and saliva genes included throughout the analysis. There are more genes at least moderately expressed in saliva (20,407 vs. 14,782), but there are significantly more differentially expressed genes in tissue between cancer and normal samples (1,302 vs. 14).

To evaluate whether saliva mirrors tissue transcriptomic profiles in GC, we independently conducted differential gene expression analysis on both tissue and saliva datasets. Following data normalization and removal of duplicates, the initial analysis included 21,367 genes, as illustrated in Fig. 2.

**Fig. 3:**
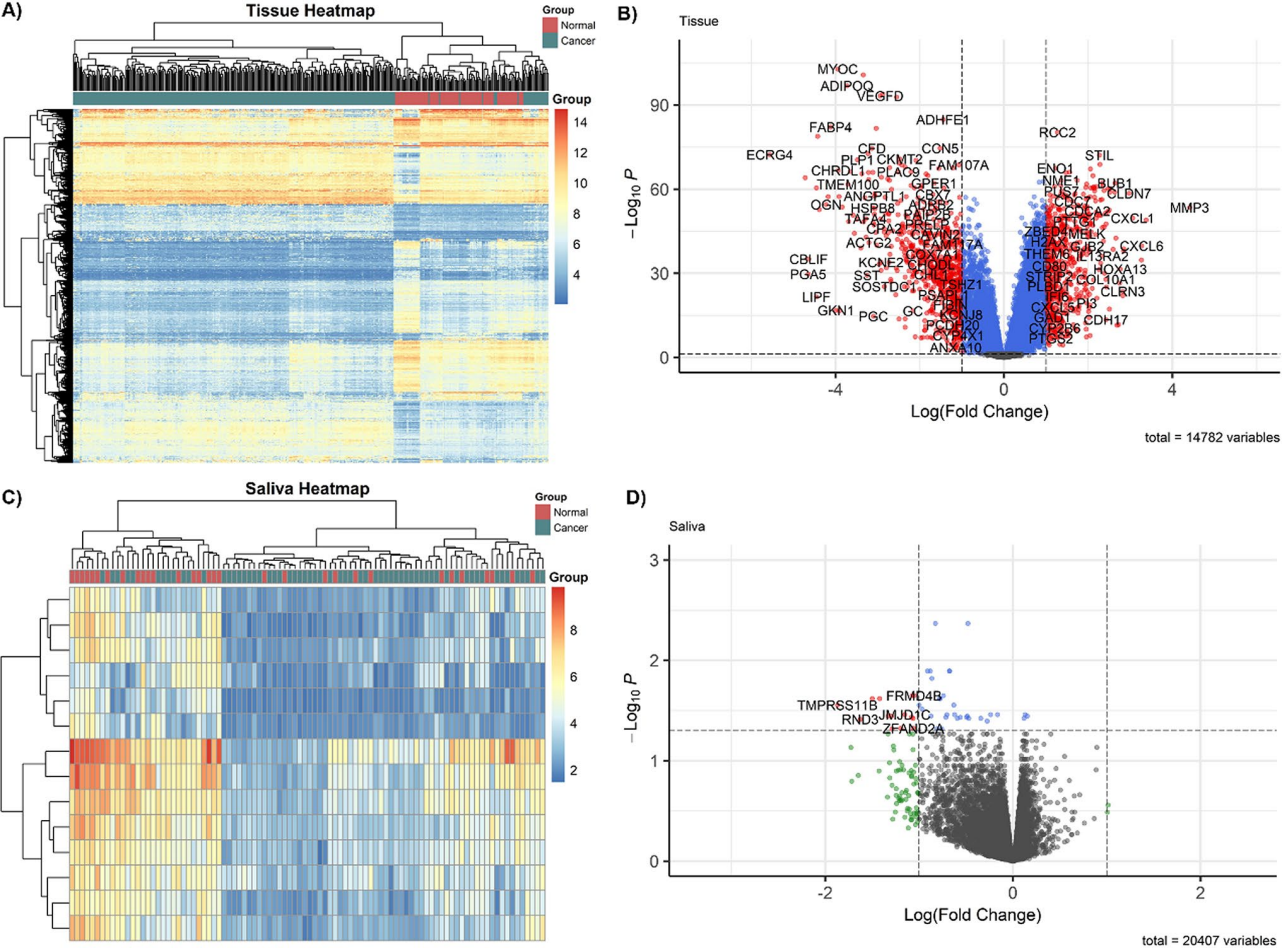
Comparative gene expression in tissue and saliva samples. (**A**) Heatmap of differentially expressed genes in tissue, distinguishing most cancer from normal samples; (**B**) Volcano plot of moderately expressed genes in tissue; (**C**) Heatmap of differentially expressed genes in saliva, without clear cancer-normal distinction; (**D**) Volcano plot of moderately expressed genes in saliva, highlighting downregulated genes in gastric cancer.

Recognizing the improved sensitivity of detecting DEGs through the removal of genes with low expression, 14,782 and 20,407 genes were retained in the tissue-derived and saliva-derived datasets, respectively^30^. Moreover, almost 98% of the genes expressed in stomach tumor tissue also presented moderate expression levels in saliva samples. Differential expression analysis revealed 1302 DEGs in tissue, with a distinct clustering pattern observed between GC and normal samples in the heatmap, as depicted in Fig. 3A. Among the highlighted DEGs in the tissue, 723 were downregulated and 579 upregulated in individuals with GC (Fig. 3B).

**Fig. 4:**
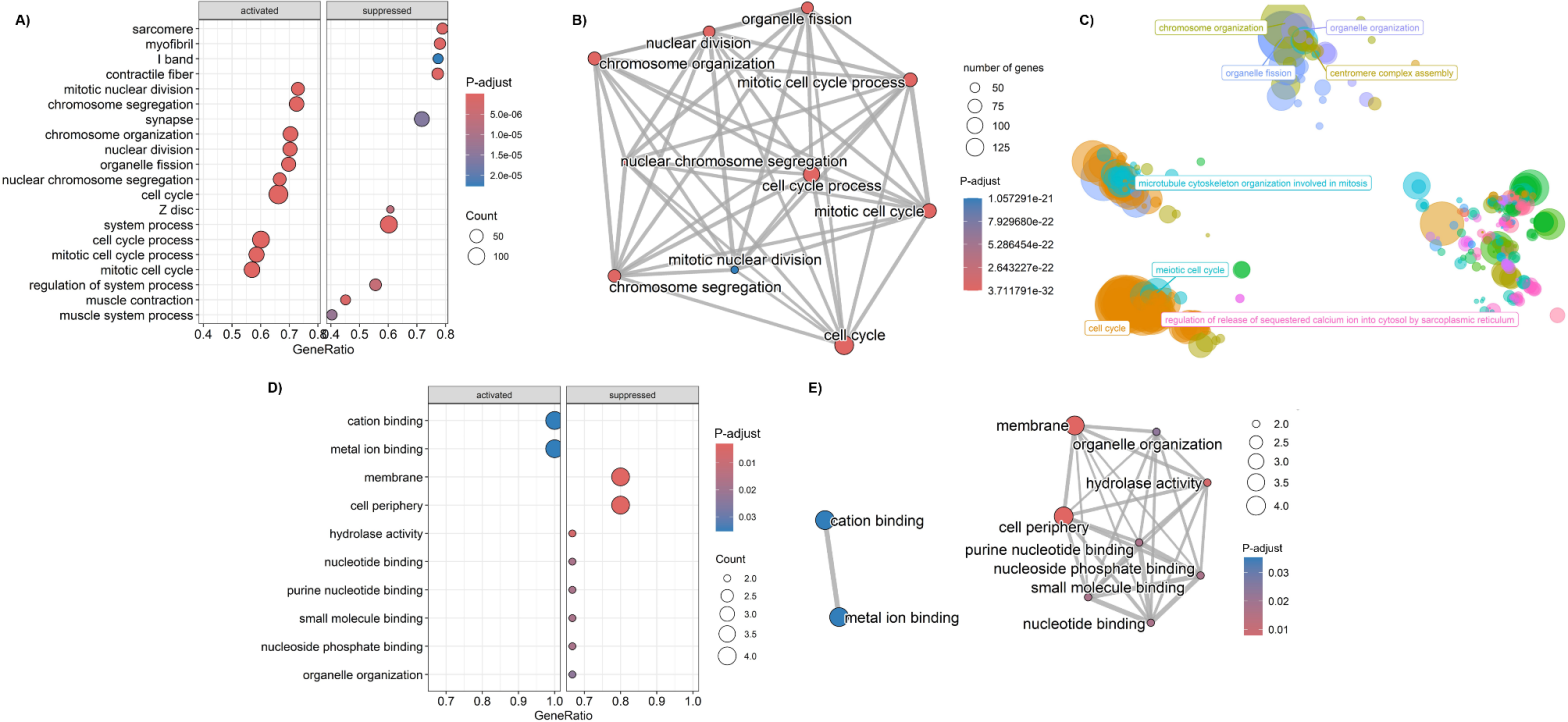
Functional enrichment analysis of differentially expressed genes (DEGs) in gastric cancer tissue (**A-C**) and saliva (**D-E**). (**A**) Dot plot showing the top enriched gene ontology (GO) terms for tissue DEGs; (**B**) Enrichment map visualizing the relationships between enriched GO terms in tissue DEGs; (**C**) Scatter plot summarizing redundant GO terms in tissue DEGs by grouping them into clusters based on semantic similarity; (**D**) Dot plot showing the top enriched GO terms for saliva DEGs; (**E**) Enrichment map visualizing the relationships between enriched GO terms in saliva DEGs.

Functional enrichment analysis revealed significant enrichment in several key biological processes, particularly those related to the cell cycle (Fig. 4A and B). In particular, “cell cycle” (normalized enrichment score (NES) = 4.54, P-adjust = 8.35 × 10^− 28^) and “mitotic cell cycle process” (NES = 4.54, P-adjust = 1.79 × 10^− 23^) were among the most enriched pathways. These findings are further summarized in the rrvgo scatter plot (Fig. 4C), which clusters and visualizes similar GO terms to reduce redundancy.

In contrast, only 14 genes were identified as differentially expressed in saliva (Fig. 3C), all downregulated in patients with GC compared with healthy controls, as illustrated in Fig. 3D. Unlike the tissue dataset, GSEA of saliva did not reveal significant enrichment in biological processes. Instead, the analysis highlighted the enrichment of cellular components and molecular functions, particularly “membrane” and “cell periphery” (NES = -2.05, P-adjust = 0.0029), “hydrolase activity” (NES = -2.05, P-adjust = 0.0125), “nucleotide binding”, “purine nucleotide binding”, and “small molecule binding” (NES = -1.85, P-adjust = 0.0335, Fig. 4D and E). The core genes driving these enrichments included *DSP*,* BAG3*,* RND3*,* TMPRSS11B*, and *SEPTIN10*. Notably, none of the DEGs identified in the saliva samples were dysregulated in the tissue dataset.

### Saliva-based molecular panel is a promising tool towards GC screening

**Fig. 5:**
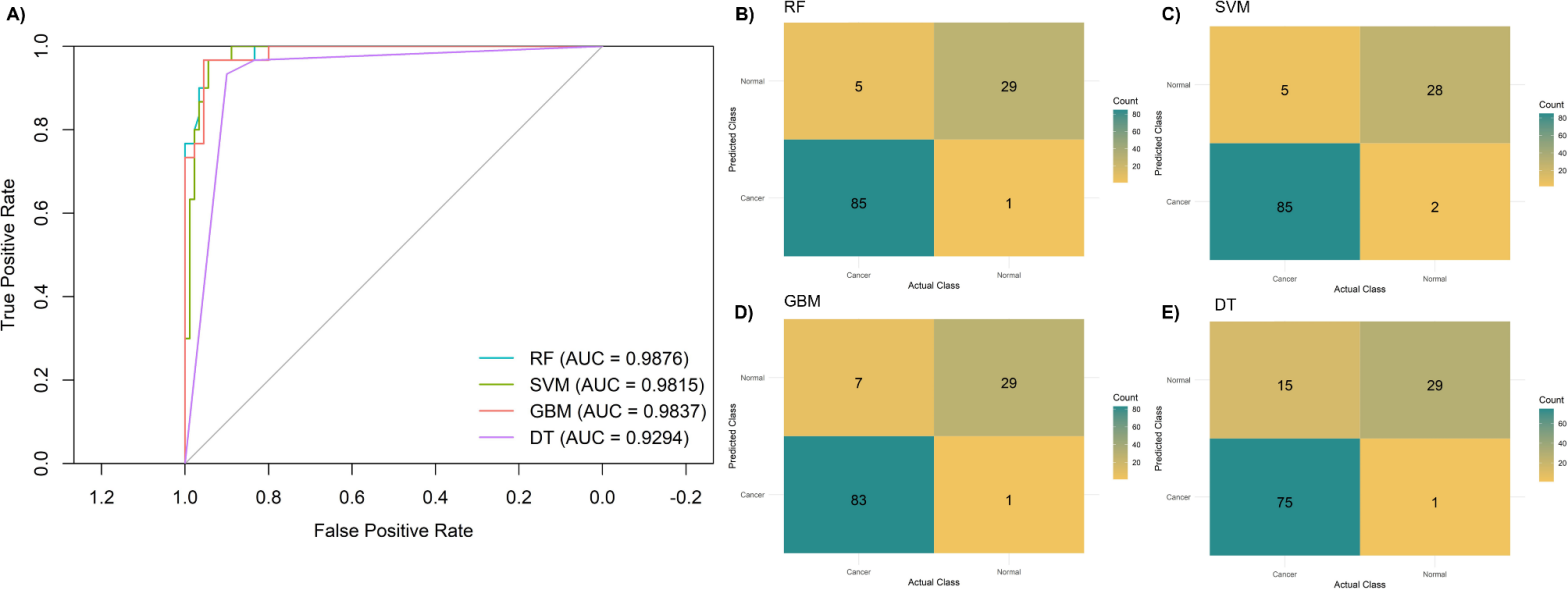
Performance of the tissue-based discriminatory models for predicting gastric cancer. (**A**) Receiver-operating characteristic (ROC) curves for random forest (RF), support vector machine (SVM), gradient boosting (GBM), and decision tree (DT). Confusion matrices showing classification performance of the tissue-based models, including (**B**) RV, (**C**) SVM, (**D**) GBM, and (**E**) DT. AUC: area under the ROC curve.

Given that saliva does not appear to mirror the transcriptomic changes observed in GC tissue, we interrogated if it could still represent a proxy for stomach malignant transformation and personalized screening. Using RF, GBM, and DT within the tidymodels framework, the *CDH3*,* DKC1*,* ESM1*,* MUSTN1*,* RCC2*,* TMT1A*, and *VEGFD* genes were selected from the tissue dataset based on their importance scores (Figure S1) and subsequently validated in the tissue discovery dataset, as summarized in Table 1. Among the models, RF exhibited the highest overall performance, with the best balance of sensitivity, specificity, and accuracy, with an AUC of 0.99 (95% confidence interval (CI): 0.97-1.00) (Fig. 5). This translates to a negative likelihood ratio (LR) of 0.06 (95% CI 0.02–0.14), and a positive LR of 28.36 (95% CI 4.16-196.63).

**Table 1:** Tissue model performance for the detection of gastric cancer.

Model	Sens (95% CI)	Spec (95% CI)	PPV (95% CI)	NPV (95% CI)	ACC (95% CI)	AUC (95% CI)
RF	0.944 (0.889–0.989)	0.967 (0.900-1.000)	0.988 (0.966-1.000)	0.853 (0.744–0.967)	0.950 (0.908–0.983)	0.988 (0.966-1.000)
SVM	0.944 (0.900-0.989)	0.933 (0.833-1.000)	0.977 (0.945-1.000)	0.848 (0.852 − 0.750)	0.942 (0.900-0.983)	0.982 (0.960–0.997)
GBM	0.922 (0.856–0.978)	0.967 (0.900-1.000)	0.988 (0.965-1.000)	0.806 (0.700-0.933)	0.933 (0.883–0.975)	0.984 (0.962–0.997)
DT	0.833 (0.744–0.911)	0.967 (0.900-1.000)	0.987 (0.961-1.000)	0.659 (0.558–0.778)	0.867 (0.800-0.925)	0.929 (0.879–0.971)

ACC, accuracy; AUC, area under the ROC curve; CI, confidence interval; DT, decision tree; GBM, gradient boosting; PPV, positive predictive value; NPV, negative predictive value; RF, random forest; Sens, sensitivity; spec, specificity; SVM, support vector machine.

**Fig. 6:**
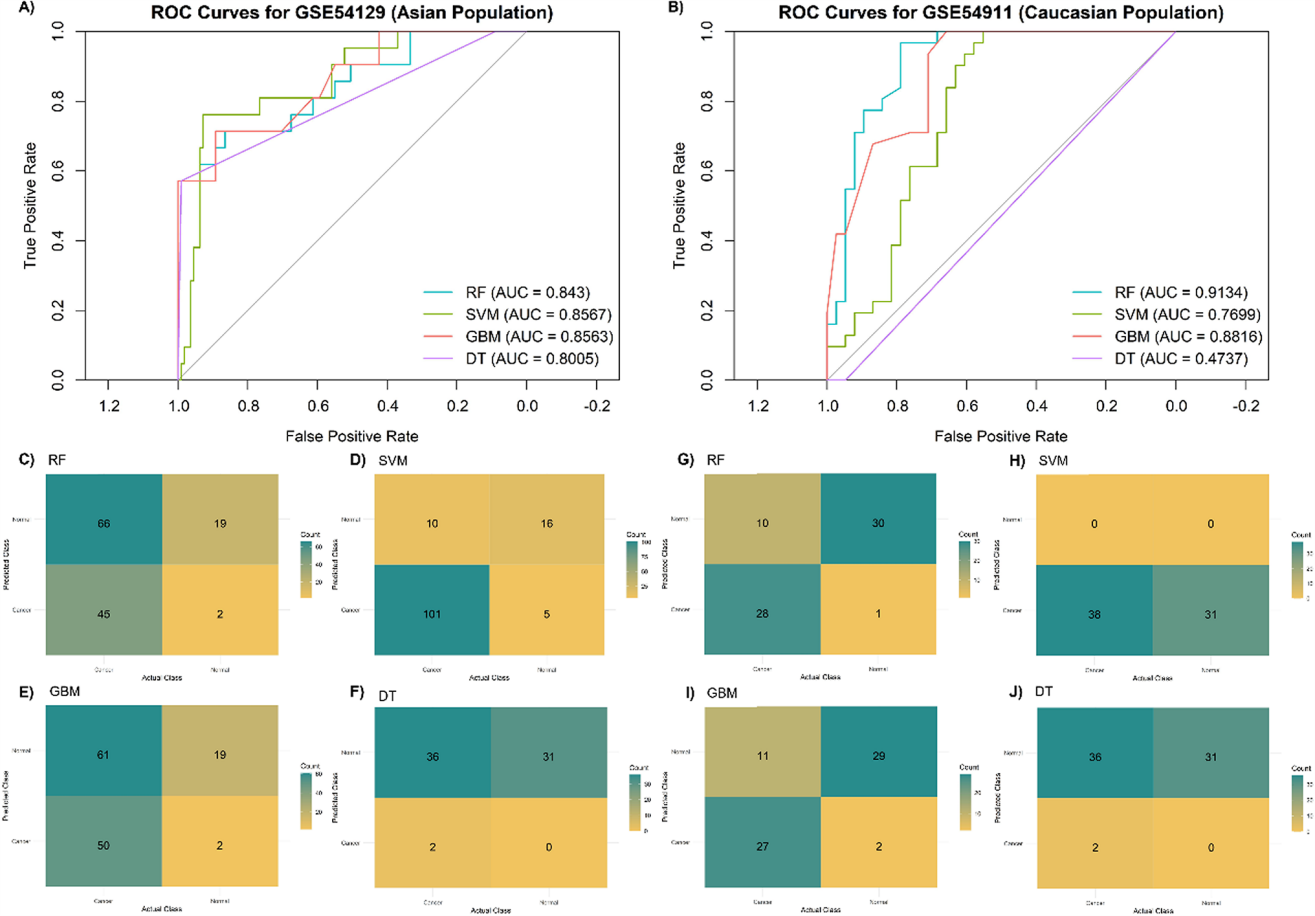
Performance of the tissue-based discriminatory models for predicting gastric cancer in the validation datasets. Receiver-operating characteristic (ROC) curves for random forest (RF), support vector machine (SVM), gradient boosting (GBM), and decision tree (DT) in the (**A**) GSE54129 (Asian) dataset and (**B**) GSE13911 (Caucasian) dataset. Confusion matrices showing classification performance of the tissue-based models using GSE54129 validation data, including (**C**) RV, (**D**) SVM, (**E**) GBM, and (**F**) DT. AUC: area under the ROC curve and using GSE13911 validation data (G-J).

The models were further tested on two independent datasets, with the performance varying considerably across datasets, as summarized in Table 2. The best performing algorithm in the Asian dataset (SVM model) demonstrated a sensitivity and accuracy of 0.91 (95% CI 0.86–0.96) and 0.89 (95% CI 0.83–0.94), respectively, with a reasonable AUC of 0.86 (95% CI 0.76–0.94), a negative LR of 0.12 (95% CI 0.06–0.22) and a positive LR of 3.82 (95% CI 1.78–8.24) (Fig. 6). On the other hand, the RF model showed the best balance in the Caucasian cohort (Fig. 6), with a sensitivity of 0.74 (95% CI 0.58–0.87), specificity of 0.97 (95% CI 0.90-1.00), and the highest AUC of 0.91 (95% CI 0.83–0.97), translating into a negative LR of 0.27 (95% CI 0.16–0.46) and a positive LR of 27.17 (95% CI 3.32-159.86).

**Table 2:** Tissue model performance for the detection of gastric cancer in the validation datasets.

ML algorithm	Sens (95% CI)	Spec (95% CI)	PPV (95% CI)	NPV (95% CI)	ACC (95% CI)	AUC (95% CI)
GSE54129 (Asian)						
RF	0.405 (0.315–0.486)	0.905 (0.762-1.000)	0.957 (0.894-1.000)	0.224 (0.186–0.259)	0.485 (0.402–0.553)	0.843 (0.725–0.935)
SVM	0.910 (0.856–0.955)	0.762 (0.571–0.952)	0.953 (0.917–0.990)	0.615 (0.480–0.783)	0.886 (0.833–0.939)	0.857 (0.757–0.935)
GBM	0.451 (0.351–0.532)	0.905 (0.762-1.000)	0.962 (0.902-1.000)	0.238 (0.195–0.276)	0.523 (0.439–0.591)	0.856 (0.757–0.943)
DT	0.090 (0.045–0.144)	1.000 (1.000–1.000)	1.000 (1.000–1.000)	0.172 (0.165–0.181)	0.235 (0.197–0.280)	0.802 (0.695–0.896)
GSE13911 (Caucasian)						
RF	0.737 (0.579–0.868)	0.968 (0.903-1.000)	0.966 (0.893-1.000)	0.750 (0.652–0.857)	0.841 (0.754–0.913)	0.913 (0.834–0.972)
SVM	1.000 (1.000–1.000)	0.000 (0.000–0.000)	0.551 (0.551–0.551)	NaN	0.551 (0.551–0.551)	0.770 (0.646–0.884)
GBM	0.711 (0.553–0.842)	0.936 (0.839-1.000)	0.931 (0.839-1.000)	0.725 (0.628–0.833)	0.812 (0.724–0.899)	0.882 (0.795–0.947)
DT	0.053 (0.000-0.132)	1.000 (1.000–1.000)	1.000 (1.000–1.000)	0.463 (0.449–0.484)	0.478 (0.449–0.522)	0.526 (0.500-0.566)

ACC, accuracy; AUC, area under the ROC curve; DT, decision tree; GBM, gradient boosting; ML, machine learning; NPV, negative predictive value; PPV, positive predictive value; RF, random forest; Sens, sensitivity; spec, specificity; SVM, support vector machine.

When applied to the liquid biopsy dataset, no machine learning model exhibited discriminatory value, each producing an AUC of 0.5, as shown in Figure S2.

**Fig. 7:**
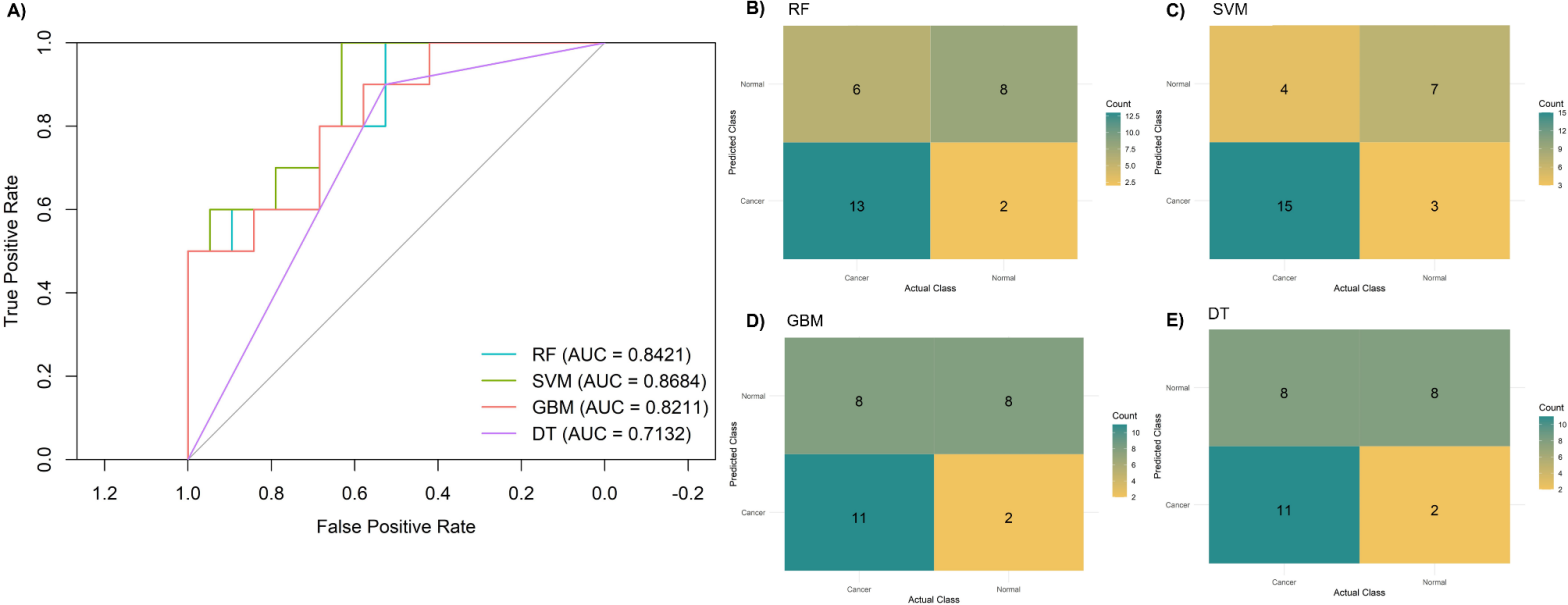
Performance of the saliva-based discriminatory models for predicting gastric cancer. (**A**) Receiver-operating characteristic (ROC) curves for random forest (RF), support vector machine (SVM), gradient boosting (GBM), and decision tree (DT). Confusion matrices showing classification performance of the saliva-based models, including (**B**) RV, (**C**) SVM, (**D**) GBM, and (**E**) DT. AUC: area under the ROC curve.

We then shifted our focus to explore the transcriptomic-derived salivary data, considering the 14 DEGs previously identified in the GSE64951 dataset: *ANKRD37*,* BAG3*,* DSP*,* FRMD4B*,* JMJD1C*,* NAA20*,* RND3*,* SDR16C5*,* SEPTIN10*,* TMPRSS11B*,* TUFT1*,* ZBED2*,* ZFAND2A*, and *ZHX1*. The performances of the RF, SVM, GBM, and DT models are summarized in Table 3. Overall, the SVM achieved the highest performance (Fig. 7), with a sensitivity of 0.79 (95% CI 0.58–0.95), specificity of 0.70 (95% CI 0.40–0.90), accuracy of 0.76 (95% CI 0.59–0.90), and AUC of 0.87 (95% CI 0.72–0.97), translating into negative and positive LRs of 0.3 (95% CI 0.1–0.92) and 2.63 (95% CI 1.17–5.91), respectively.

**Table 3:** Saliva model performance for detecting gastric cancer.

Model	Sens (95% CI)	Spec (95% CI)	PPV (95% CI)	NPV (95% CI)	ACC (95% CI)	AUC (95% CI)
RF	0.684 (0.474–0.895)	0.800 (0.500-1.000)	0.867 (0.722-1.000)	0.571 (0.421-0.800)	0.724 (0.586–0.897)	0.842 (0.679–0.963)
SVM	0.790 (0.579–0.947)	0.700 (0.400–0.900)	0.833 (0.700-0.947)	0.636 (0.429–0.889)	0.759 (0.586–0.897)	0.868 (0.721–0.974)
GBM	0.579 (0.368–0.789)	0.800 (0.500-1.000)	0.846 (0.692-1.000)	0.500 (0.368–0.692)	0.655 (0.483–0.828)	0.821 (0.637–0.958)
DT	0.526 (0.316–0.737)	0.900 (0.700-1.000)	0.909 (0.750-1.000)	0.500 (0.391–0.667)	0.655 (0.483–0.828)	0.713 (0.579–0.845)

ACC, accuracy; AUC, area under the ROC curve; DT, decision tree; GBM, gradient boosting; PPV, positive predictive value; NPV, negative predictive value; RF, random forest; Sens, sensitivity; spec, specificity; SVM, support vector machine.

Table 4 summarizes the best model performance across tissue and saliva datasets, comparing sensitivity, specificity, and AUC values.

**Table 4:** Comparison of the best model performance across tissue and saliva datasets.

	Model	Sens (95% CI)	Spec (95% CI)	AUC (95% CI)
Tissue (GSE66229)	RF	0.944 (0.889–0.989)	0.967 (0.900-1.000)	0.988 (0.973-1.000)
Tissue (GSE54129 – Asian)	SVM	0.910 (0.856–0.955)	0.762 (0.571–0.952)	0.857 (0.757–0.935)
Tissue (GSE13911 – Caucasian)	RF	0.737 (0.579–0.868)	0.968 (0.903-1.000)	0.913 (0.834–0.972)
Saliva (GSE64951)	Saliva SVM	0.790 (0.579–0.947)	0.700 (0.400–0.900)	0.868 (0.721–0.974)

AUC, area under the ROC curve; CI, confidence interval; RF, random forest; Sens, sensitivity; spec, specificity; SVM, support vector machine.

## Discussion

While saliva does not mirror the transcriptomic landscape observed in stomach tumorous tissue, it represents a promising non-invasive tool in GC screening and early detection.

Traditional tissue sampling for histopathological analysis offers the advantage of establishing short- and long-term management strategies through comprehensive assessment of the gastric mucosa^31^. However, this approach has significant drawbacks, requiring invasive, time-consuming endoscopic procedures that demand a high level of skill and experience^31^. Despite providing a clear image of the gastric mucosa and enabling the collection of tissue samples, these limitations render it suboptimal for widespread screening of the general population^31^. In contrast, saliva has emerged as a non-invasive biofluid that is easily collected, stored, and transported^32^.

Despite being pointed out as a mirror of health state, few studies have compared the transcriptomic profiles between tissues and saliva beyond comparisons with gingival tissue biopsies in periodontitis^33^. Challenges, such as the low abundance of biomarkers and the difficulties in collecting large saliva volumes, contributed to the scarcity of published data^34^. The evolving omics field, fuelled by technological advancements, has revolutionized biomedical research by allowing cost-effective high-throughput analyses, with extensive datasets of mRNA, microRNA, protein, and genetic information, conserving resources and minimizing sample waste^35–37^.

Using publicly available data, we conducted a comparative analysis of the transcriptomic landscape between tissue and saliva samples in GC. Notably, we observed a significantly greater number of genes that were at least moderately expressed in saliva than in tissue (20407 vs. 14782). This discrepancy likely stems from the contributions of the entire organism to the salivary transcriptomic profile, as opposed to tissue, where the primary contributors are stomach cells. This underscores saliva as a highly rich liquid biopsy, representing a valuable source of non-invasive biomarkers.

Using machine learning algorithms, including RF, SVM, GBM (XGBoost), and DT, the genes *CDH3*,* DKC1*,* ESM1*,* MUSTN1*,* RCC2*,* TMT1A*, and *VEGFD* were selected as key features from the tissue dataset to best discriminate between cancer and control samples and were used to design the predictive model. This transcriptomic signature showed excellent discriminatory power, correctly identifying positive and negative cases with high accuracy (AUC > 0.9), across all machine learning methods employed. However, upon validation, the performance of these models varied between different population datasets. In the Asian dataset (GSE54129), the SVM model showed better performance, reaching an AUC value of 0.857 with over 0.90 sensitivity and 0.76 specificity. In our study, a low positive or high negative LR (closer to zero) is desirable for ruling out GC, whereas a high positive LR (greater than 10) is good for confirming it. In the SVM model applied to the Chinese population, a negative LR of 0.12 indicates a moderate decrease in the probability of GC being present, whereas a positive LR value of 3.82 suggests a small increase in the likelihood of disease. In contrast, within the Caucasian dataset, the RF model outperformed the other algorithms, demonstrating higher specificity (0.97), moderate sensitivity (0.74) and an AUC of 0.91. This translates to a negative LR of 0.27, indicating a small decrease in the probability of GC, and a positive LR of 27.17, reflecting a substantial increase in the likelihood of disease. This variation in model performance across different populations suggests that population-specific factors – such as genetic differences, environmental influences, and disease heterogeneity – may influence the effectiveness of the predictive model, highlighting the importance of considering genetic diversity in model validation. Additionally, technical biases, such as batch effects and differences in sample processing methods, could have contributed to the observed performance discrepancies between the validation datasets. Although normalization using the RMA method and stratified sampling were employed to mitigate potential biases in both training and validation datasets, these inherent dataset-specific factors highlight the importance of considering genetic diversity and technical variation when validating machine learning models for clinical applications. Further, larger-scale validation datasets from diverse geographical regions are needed to assess the generalizability and robustness of these models in different population groups.

The mixed fluid flowing into the oral cavity originates predominantly from intercellular fluid secreted by the salivary glands, and blood^38^. Consequently, various substances from blood that are transported through transcellular routes, such as active transport and passive diffusion, or paracellular routes, such as extracellular ultrafiltration, can be detected in saliva^39^. Specifically, tumor-secreted exosomes, which carry tumor-derived proteins, RNA, and other biomolecules, can be transported via systemic circulation and eventually be secreted into saliva^40^. Additionally, the systemic inflammatory response triggered by gastric malignancy can lead to changes in the composition of salivary proteins and nucleic acids, potentially reflecting the underlying disease process^41^. Changes in the molecular landscape of tissue can lead to alterations in the transcriptomic profiles of blood and, subsequently, oral fluid^38^. However, variations in biomarker transfer mechanisms may result in distinct molecular compositions across different matrices^38^. Notably, no common DEGs were identified between the tissue and saliva datasets under study, and we were unable to discriminate between classes in the saliva dataset via any of the tissue-derived models. This observation is consistent with findings from other studies, such as studies on stool samples, which have shown greater sensitivity to tissue changes, particularly in colorectal cancer, than in blood^42^. For example, among the differentially expressed microRNAs in stool, 56% demonstrated concordant expression in colorectal tumor tissue, whereas only 8% demonstrated commonality between stool and plasma extracellular vesicles, as reported in^42^.

Following the inability of the tissue-derived model to distinguish between cancer and control samples in the saliva dataset, we developed a new predictive model based exclusively on salivary transcriptomic data. Leveraging the 14 DEGs identified in the GSE64951 dataset, we explored the potential of this liquid biopsy as a proxy for the non-invasive detection of GC. Although the best-performing salivary model, SVM, did not match the excellent accuracy of the tissue-based model, it demonstrated good performance, achieving an AUC of 0.87. Additionally, it yielded a sensitivity and specificity of 0.79 and 0.70, respectively, translating into a negative LR of 0.3, which suggests a modest ability to rule out GC when the test is negative, and a positive LR of 2.63, pointing to a small increase in the likelihood of disease when the test is positive. Thus, while the performance of the salivary signature is not as good as that of the tissue model, the non-invasive nature of saliva collection offers a clear practical advantage. Moreover, comparing this model to the serum pepsinogen test, a standard screening tool for GC, highlights its potential. The salivary model demonstrated a trend towards higher sensitivity (0.79, 95% CI 0.58–0.95, vs. 0.69, 95% CI 0.60–0.76) and comparable specificity (0.70, 95% CI 0.40–0.90, vs. 0.73, 95% CI 0.62–0.82), suggesting that saliva could be a competitive option for non-invasive screening^43^. Moreover, it seems to be more effective at ruling out the condition with a lower negative LR (0.30, 95% CI 0.1–0.92, vs. 0.43, 95% CI 1.17–5.91), although with no confirmation of statistical significance, while demonstrating a similar ability to confirm the disease when the test is positive (positive LR of 2.63, 95% CI 1.17–5.91 vs. 2.57, 95% CI 1.82–3.62)^43^. Given that the pepsinogen test typically falls within the fair AUC range, the saliva model demonstrates encouraging performance that warrants further exploration.

This study has a few limitations. Despite the considerable potential of saliva as a liquid biopsy for disease screening and diagnosis, the paucity of publicly available data remains a challenge. To the best of our knowledge, only two saliva databases on GC were available from GEO, one of which was analysed in this study (GSE64951), and the other, encompassing noncoding RNA profiling by high-throughput sequencing (GSE121870), lacked raw data accessibility. The smaller sample size of the dataset limits the statistical power of our findings. Furthermore, and although both from Asian origin (Korean), the tissue and saliva samples were collected from different populations. Another key limitation was the lack of clinical data available in the GEO repository, which hindered further exploration of variables such as tumor staging, age, and other clinical variables relevant to GC development, which could influence model performance, acting as confounding factors, and limit the generalizability of the findings. Additionally, we recognize the challenges associated with standardizing saliva collection and processing protocols. Variability in sample handling, storage, and processing could significantly impact biomarker consistency and reproducibility, which is crucial for clinical implementation. From a biological perspective, we were unable to obtain significant results when performing GSEA in saliva, indicating that these genes do not exhibit common pathway involvement in GC. This limitation restricts our ability to directly link these biomarkers to specific biological processes or pathways in GC. Further research should prioritize larger, independent, and multicentric studies that include diverse populations and detailed clinical metadata to enhance the robustness of salivary biomarker panels. Standardizing saliva collection and processing protocols is also essential to improve reproducibility. Additionally, longitudinal studies could help determine the clinical feasibility of saliva-based screening. While our findings suggest that saliva may not fully capture the transcriptomic changes occurring in gastric tumor tissue in these datasets, it remains an accessible and promising bodily fluid for non-invasive diagnosis. Future research should aim to optimize liquid biopsy strategies and explore whether other fluids, such as plasma or gastric juice, or molecular markers may serve as more reliable proxies for tissue-based alterations in GC. With circulating biomarkers emerging as promising alternatives to traditional methods, their integration into personalized medicine practices has the potential to revolutionize early detection strategies, providing a more patient-centric and precise approach to disease management in the era of personalized medicine.

## Electronic supplementary material

Below is the link to the electronic supplementary material.

Supplementary Material 1

## Data Availability

The datasets supporting the conclusions of this article are available in the Gene Expression Omnibus at https://www.ncbi.nlm.nih.gov/geo/. These data were derived from the following resources available in the public domain: - GSE13911, https://www.ncbi.nlm.nih.gov/geo/query/acc.cgi? acc=GSE13911- GSE54129, https://www.ncbi.nlm.nih.gov/geo/query/acc.cgi? acc=GSE54129- GSE64951, https://www.ncbi.nlm.nih.gov/geo/query/acc.cgi? acc=GSE64951- GSE66229, https://www.ncbi.nlm.nih.gov/geo/query/acc.cgi? acc=GSE66229.

## Abbreviations

AUC: Area under the curve
CI: Confidence interval
DEG: Differentially expressed gene
FC: Fold change
GC: Gastric cancer
GEO: Gene Expression Omnibus
LR: Likelihood ratio
NPV: Negative predictive value
PPV: Positive predictive value
ROC: Receiver operating characteristic
