# Supplementary material for: Saliva-derived transcriptomic signature for gastric cancer detection using machine learning and leveraging publicly available datasets
Source: Sci Rep. 2025 May 27;15:18491. doi: 10.1038/s41598-025-96864-0 (PMC12116728; doi:10.1038/s41598-025-96864-0)
Supplement: Supplementary file 1 — Supplementary Material 1 [file 41598_2025_96864_MOESM1_ESM.docx]

**Supplementary data**

**Table S1.** R packages used for data analysis, including version numbers and sources.

| **Package Name** | **Version** | **Source** |
| --- | --- | --- |
| limma | 3.58.1 | Bioconductor (https://bioconductor.org/packages/limma) |
| clusterProfiler | 4.10.1 | Bioconductor (https://bioconductor.org/packages/clusterProfiler) |
| DOSE | 3.28.2 | Bioconductor (https://bioconductor.org/packages/DOSE) |
| rrvgo | 1.14.2 | Bioconductor (https://bioconductor.org/packages/rrvgo) |
| affy | 1.80.0 | Bioconductor (https://bioconductor.org/packages/affy) |
| tidymodels | 1.3.0 | CRAN (https://cran.r-project.org/package=tidymodels) |
| rsample | 1.2.1 | CRAN (https://cran.r-project.org/package=rsample) |
| recipes | 1.1.1 | CRAN (https://cran.r-project.org/package=recipes) |
| themis | 1.0.3 | CRAN (https://cran.r-project.org/package=themis) |
| parsnip | 1.3.1 | CRAN (https://cran.r-project.org/package=parsnip) |
| tune | 1.3.0 | CRAN (https://cran.r-project.org/package=tune) |
| dials | 1.4.0 | CRAN (https://cran.r-project.org/package=dials) |
| vip | 0.4.1 | CRAN (https://cran.r-project.org/package=vip) |
| yardstick | 1.3.2 | CRAN (https://cran.r-project.org/package=yardstick) |
| pROC | 1.18.5 | CRAN (https://cran.r-project.org/package=pROC) |


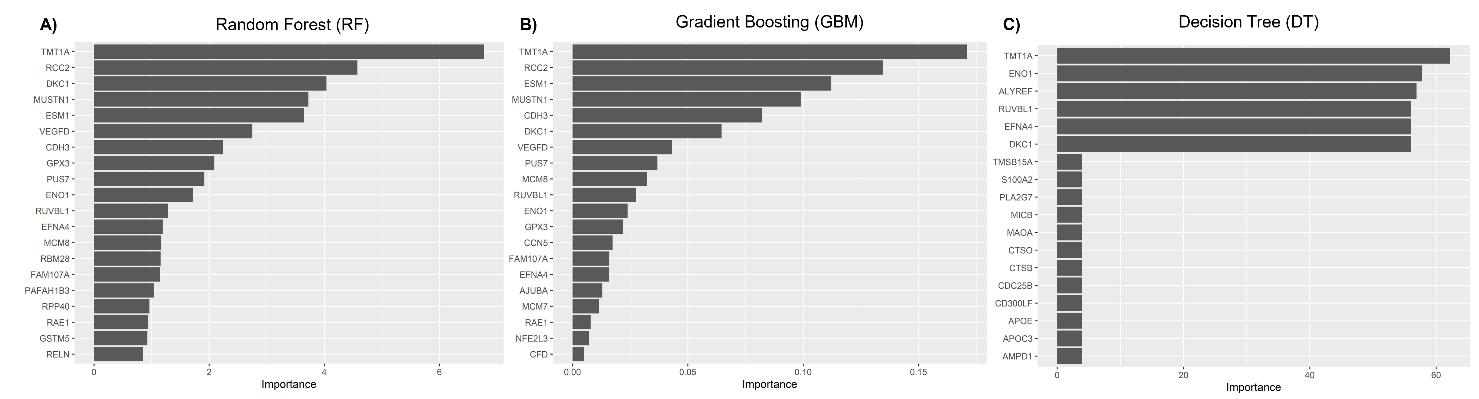


**Figure S1.** Selection of best features for building a discriminatory model using the tissue dataset using A) random forest (RF), B) gradient boosting (GBM), and C) decision tree (DT).


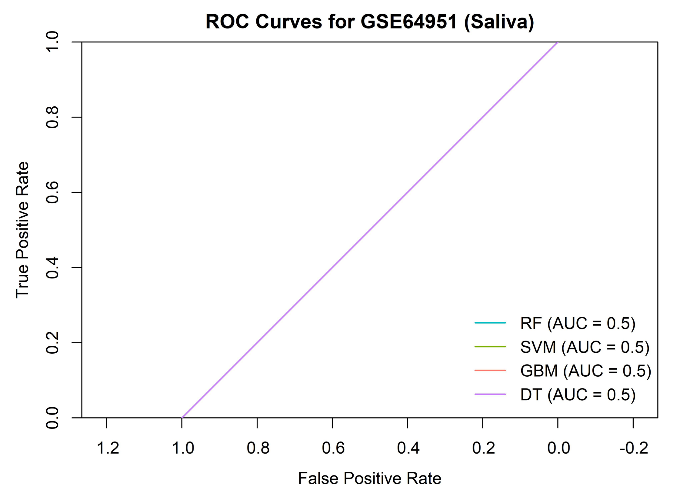


**Figure S2. Performance of the tissue-based discriminatory models for predicting gastric cancer in the saliva dataset.** Receiver-operating characteristic (ROC) curves for random forest (RF), support vector machine (SVM), gradient boosting (GBM), and decision tree (DT).
